# Hybrid Nanofibrous Membranes as a Promising Functional Layer for Personal Protection Equipment: Manufacturing and Antiviral/Antibacterial Assessments

**DOI:** 10.3390/polym13111776

**Published:** 2021-05-28

**Authors:** Latifah Abdullah Alshabanah, Mohamed Hagar, Laila A. Al-Mutabagani, Ghada M. Abozaid, Salwa M. Abdallah, Nader Shehata, Hoda Ahmed, Ahmed H. Hassanin

**Affiliations:** 1 Chemistry Department, College of Science, Princess Nourah Bint Abdulrahman University, Riyadh 11671, Saudi Arabia; laalsabanah@pnu.edu.sa (L.A.A.); laalmutbagani@pnu.edu.sa (L.A.A.-M.); 2 Chemistry Department, College of Sciences, Taibah University, Yanbu 30799, Saudi Arabia; ahoda@sci.cu.edu.eg; 3 Chemistry Department, Faculty of Science, Alexandria University, Alexandria 21321, Egypt; 4 Pharmaceutical Practice Department, College of Pharmacy, Princess Nourah Bint Abdulrahman University, Riyadh 11671, Saudi Arabia; Gaabozeed@pnu.edu.sa; 5 Mammalian and Aquatic Toxicology Department, Central Agricultural Pesticides Lab (CAPL), Agricultural Research Center (ARC), Giza 12611, Egypt; salwaabdallah17@gmail.com; 6 Center of Smart Materials Nanotechnology and Photonics (CSMNP), Smart CI Research Center, Alexandria University, Alexandria 21544, Egypt; nader83@vt.edu (N.S.); ahassanin2003@yahoo.com (A.H.H.); 7 Department of Engineering Mathematics and Physics, Faculty of Engineering, Alexandria University, Alexandria 21544, Egypt; 8 USTAR Bio Innovations Center, Faculty of Science, Utah State University, Logan, UT 84341, USA; 9 Department of Physics, School of Engineering, Kuwait College of Science and Technology (KCST), Doha Superior Rd., Jahraa 13133, Kuwait; 10 Department of Chemistry, Faculty of Science, Cairo University, Cairo 12613, Egypt; 11 Materials Science & Engineering Department, School of Innovative Design Engineering, Egypt-Japan University of Science and Technology (E-JUST), New Borg El-Arab City, Alexandria 21934, Egypt; 12 Department of Textile Engineering, Faculty of Engineering, Alexandria University, Alexandria 21544, Egypt

**Keywords:** nanofibers (PVA and TPU), Ag nanoparticles, antiviral-antibacterial, COVID-19

## Abstract

In this research work, nanofibrous hybrids are manufactured, characterized, and assessed as active antiviral and antibacterial membranes. In more detail, both polyvinyl alcohol (PVA) and thermoplastic polyurethane (TPU) nanofibrous (NF) membranes and their composites with embedded silver nanoparticles (Ag NPs) are manufactured by an electrospinning process. Their morphological structures have been investigated by a scanning electron microscope (SEM) which revealed a homogenous distribution and almost beads-free fibers in all manufactured samples. Characterization with spectroscopic tools has been performed and proved the successful manufacturing of Ag-incorporated PVA and TPU hybrid nanofibers. The crystalline phase of the nanofibers has been determined using an X-ray diffractometer (XRD) whose patterns showed their crystalline nature at an angle value (2θ) of less than 20°. Subsequent screening of both antiviral and antibacterial potential activities of developed nanohybrid membranes has been explored against different viruses, including SARS-Cov-2 and some bacterial strains. As a novel approach, the current work highlights potential effects of several polymeric hybrids on antiviral and antibacterial activities particularly against SARS-Cov-2. Moreover, two types of polymers have been tested and compared; PVA of excellent biodegradable and hydrophilic properties, and TPU of excellent mechanical, super elasticity, hydrophobicity, and durability properties. Such extreme polymers can serve a wide range of applications such as PPE, filtration, wound healing, etc. Consequently, assessment of their antiviral/antibacterial activities, as host matrices for Ag NPs, is needed for different medical applications. Our results showed that TPU-Ag was more effective than PVA-Ag as HIV-1 antiviral nanohybrid as well as in deactivating spike proteins of SARS-Cov-2. Both TPU-Ag and PVA-Ag nanofibrous membranes were found to have superior antimicrobial performance by increasing Ag concentration from 2 to 4 wt.%. Additionally, the developed membranes showed acceptable physical and mechanical properties along with both antiviral and antibacterial activities, which can enable them to be used as a promising functional layer in Personal Protective Equipment (PPE) such as (surgical gowns, gloves, overshoes, hair caps, etc.). Therefore, the developed functional membranes can support the decrease of both coronavirus spread and bacterial contamination, particularly among healthcare professionals within their workplace settings.

## 1. Introduction

The properties of materials at the nanoscale size significantly differ of those on a macroscopic scale, which becomes the origin of nanoscience development at the crossroads of physics, chemistry, and biology [1,2]. Research in the fields of both synthesis and characterization of nanomaterials constitutes a vast field of investigation today. Among the promising methods of preparing nanostructures, electrospinning is a technique used for the production of nanofibers which depends on electrostatic spinning methodology [3,4]. This process allows for the production of fibrous nonwoven or filaments of variable diameters and, consequently, controlled physical properties [5,6]. There are multiple unique properties of such generated nanofiber mats including higher surface/volume ratios, controllable interfibrous porosity, and larger adsorption capacity [7]. Electrospun nanofibers are promising candidates for many applications such as sensors [8], electronics [9], and biomedical field [10]. There are several methods of the electrospinning for generating new functional hybrids like, single-fluid process [11], coaxial [12], tri-axial, modified triaxial, side-by-side [13], multiple-fluid [14], and solid needle and needleless processes. These methods are mainly describing how to guide the polymer solutions into the electrical fields in elaborate manners for creating polymer-based nanohybrids. However, nano-suspensions of filament-forming polymers containing inorganic nanoparticles can extend the capability of electrospinning in generating new functional hybrids.

In a correlated track, metal aggregates of nanometric size have been known and exploited for centuries according to their spectacular properties [15,16]. Noble or precious metals including silver (Ag), gold (Au), and copper (Cu) (less noble) are chemical elements of extensive usage in different applications [17,18]. The interest of studying this type of metals mainly lays to their exceptional physicochemical (electrical, optical, thermal, electronic, and biological) properties. Silver is considered one of the most important elements due to its relatively reasonable cytotoxicity level along with its promising performance as an anti-bacterial agent [19,20,21]. On the other hand, hybrid materials based on polymers embedded with Ag nanoparticles are receiving considerable attention due to their superior characteristics, such as optical, electrical catalytic, and thermal properties [16,17,18,19]. These properties strongly depend on both size and shape of the prepared nanoparticles. Therefore, it would be useful to control these parameters during the synthesis process according to the envisaged applications, such as antibacterial/antiviral activities and infections-resistance, where an infection is called to be nosocomial when it is contracted in hospitals [22].

From the safety point-of-view, usage of AgNPs has multiparameters that control its toxicity, including its physicochemical properties, exposure route, dose, and duration. As human exposure to AgNPs embedded in the PPE may take place through the skin—the first line of defense with the external environment—the potential of NPs to penetrate healthy and breached human skin, as well as their ability to diffuse into underlying structures, has been well demonstrated [23,24]. Due to its potent antibacterial effect, AgNP is considered an excellent wound dressing and effective skin topical antibacterial agent with safe impacts on human health if used in reasonable quantities [25,26,27]. However, it is worthy to state that there are still gaps in the risk assessment of the Ag in the form of NPs both for humans and the environment [28]. Future fabricated antiviral/antimicrobial agents should be highly effective at low doses, and extremely durable which in turn, will help to reduce the quantities of textiles waste disposal and improve the overall carbon footprint associated with protective clothing.

Moving to the infection-resistance point, essentially there are of two types infections; either directly linked to a treatment or related to the hospital environment [29]. Healthcare-related nosocomial infections are caused by a microbe at a normally sterile site in the body, for example during surgery which is called an invasive procedure [30]. Therefore, the risk of the breakdown of first natural defenses against infection allows the inoculation of germs into deep tissues or organs. For the nosocomial infections which are linked to the hospital environment, the microbes are presented in the environment (water, air, dust, other patients, and staff themselves infected) could infect the patient through the respiratory, digestive tract, or by contact [31]. This is, for example, legionellosis conveyed by the air conditioning system, the water network, or aspergillosis (a fungus) whose spores are suspended in the air and possibly could be inhaled by people. People themselves who are infected by tuberculosis, influenza, Southeast Asian pneumonia, or SARS can transmit their infections to other patients. Different pieces of evidence include promiscuity, frequency of contacts, presence of patients suffering from infectious diseases, and patients vulnerable to infections (elderly or immunocompromised) can prove that the hospital is conductive to the transmission of such infections. Furthermore, staff can receive an infection through the respiratory tract (tuberculosis, SARS) or during accidental exposure to the blood of a patient infected with hepatitis B or C or with HIV). Therefore, they can be another source of infection transfer to contacted patients [32].

The aim of this work is to develop an antiviral/antibacterial membrane from electrospun biodegradable and synthetic nanofibers that could be used as a promising functional layer in personal protection equipment (PPE), such as gowns and others hospitals equipment. Herein, the developed nanofibers, TPU and PVA loaded with Ag nanoparticles, have been characterized and investigated for their potential antiviral and antibacterial activities as well. The new contribution here is to offer a competitive study between the effect of the polymeric host type on both antiviral and antibacterial activities, especially on SARS-COV-2.

## 2. Material and Methods

### 2.1. Manufacturing of Nanofibrous Membranes

Polyvinyl alcohol (PVA) pellets (Mw = 205,000 g/mol, Sigma Aldrich, St. Louis, MO, USA) were used to prepare 10 wt.% of PVA solution. The PVA pellets were added to distilled water at 70 °C for 1 h then left on a stirrer overnight at room temperature. Thermoplastic polyurethane (TPU) with Polydispersity Index (PDI) of 1.83 and 107,000 g/mol molecular weight was supplied by (BASF Co., Ltd., Berlin, Germany). TPU solution was prepared by dispersing TPU pellets in dimethyl formamide (DMF) with stirring for 24 h to attain 10 wt.% of the solution. The Ag NPs were prepared as (0.05 M) of AgNO_3_ aqueous solution and (0.01 M) glucose are mixed by adding drops of 25% ammonia solution in order to adjust pH to 8. Glucose was used as a reducing agent for the reduction of Ag^+^ ions into Ag metal and Ag NPs [33]. The size of AgNPs was obviously large coarse powder so that reducing the size of AgNPs is required for nanofiber applications. Consequently, AgNPs were milled using ball mill (PM100; Retsch) to get fine powder. Periodically, each 1.4 g of AgNPs was milled using 10 balls (10 mm) at 150 rpm for 3 h. Different spectroscopic instruments at the Central Lab of faculty of science, Alexandria were used to analyze the prepared powder. The particle size is 17–51 nm from its spectral data of Ag nanoparticles, (see supplementary data, Figures S1–S5). Ag nanoparticles were added with different concentration [34] (2 wt.%, 4 wt.%) to the prepared solutions and left on stirrer overnight. The prepared solutions were electrospun by adding 5 mL of each concentration into a plastic syringe with gauge 18 stainless-steel needle. High voltage power supply CZE1000R (Spellman, Hauppauge, NY, USA) was connected and provided positive volt age of 25 kV to the needle. A feed rate of 1 mL/h was fixed by using a syringe pump NE1000 (New Era Pump Systems, Suffolk County, NY, USA). The distance between the needle tip and grounded rotating collector was adjusted to 10 cm.

### 2.2. Fiber Morphology

The morphological structures of PVA and TPU NFs have been observed by the Scanning Electron Microscope (JEOL, JSM-6010LV-SEM). One sample for each pressure was cut and stacked onto carbon tabs before sputtering with platinum. The average fiber diameter was measured using Image-J software at three different image scales (1, 5, and 10 µm).

### 2.3. Physical Characterization

The crystalline phase of PVA, TPU, and electrospun NFs were determined using X-Ray Diffractometer (XRD) (Shimadzu Xlab 6100 instrument using Cu as a target), scanning range of 5–80° and scanning speed of 1 deg/min for precise detection of peaks. Fourier transform infrared spectrometer (FT-IR) (Vertex 70 FT-IR, Bruker, Billerica, MA, USA) was operated in ATR mode. Samples were scanned 120 times at a resolution of 5 cm^−1^ over a range of 4000–400 cm^−1^.

### 2.4. Antiviral Activity of the Prepared Nanofibers

The anti-COVID-19 activity of the manufactured nanofibers (PVA-Ag and TPU-Ag) was evaluated using SARS-Cov-2 inhibitor screening ELISA kit (COVID-19 Coronavirus Assay Kit, BioSource, Muskego, WI, USA) that measures the binding of the RBD of the Spike S protein from SARS-Cov-2 virus to its human receptor ACE2. Different concentrations of the nanofibers were prepared and incubated with Spike S proteins for 1 h at 37 °C then the OD was measured at 450 nm using ELISA reader [35]. A similar test was evaluated against HIV (HIV-1 Protease Inhibitor Screening Kit, BioVision, San Francisco, CA, USA) in order to evaluate the antiviral activity of PVA-Ag and TPU-Ag against various RNA viruses. The fluorescence was measured (330/450 nm) in a kinetic mode for 1–3 h at 37 °C. Relative inhibition of all tested nanofibers (S) and Enzyme Control (EC) was calculated using the following equation:

(1) $$\%\mathrm{Relative}\;\mathrm{inhibition}\;=\frac{\mathrm{Slope}\;\mathrm{of}\;\mathrm{EC}-\mathrm{Slope}\;\mathrm{of}\;S}{\mathrm{Slope}\;\mathrm{of}\;\mathrm{EC}}\times 100$$

### 2.5. Antibacterial Activity of the Prepared Nanofibers

The antibacterial activity of different manufactured nanofibers was evaluated via various methods. An aliquot of each bacterial strain (10^6^ CFU/mL) was spread onto Muller Hinton agar (MHA) plates and then different nanofibers of fixed dimensions (1 cm × 1 cm) were overlaid onto the inoculated agar. The plates were then incubated for 24 h at 37 ± 2 °C. The antibacterial activity was expressed as inhibition zone halos around the tested nanofibers [23]. Antibacterial kinetics of PVA-Ag and TPU-Ag nanofibers (1 cm × 1 cm) were evaluated using 50 mL of bacterial suspension with different concentrations (from 10^2^ CFU/mL to 10^10^ CFU/mL). After 24 h of incubation, the findings were studied using SEM to determine the antibacterial mechanism of the tested nanofibers [23].

Results were expressed as mean of three trials ± standard deviation. The means of the treatments were considered significant when 0.05 > *p* > 0.01.

## 3. Results and Discussion

### 3.1. Morphological Characterization

**Figure 1:**
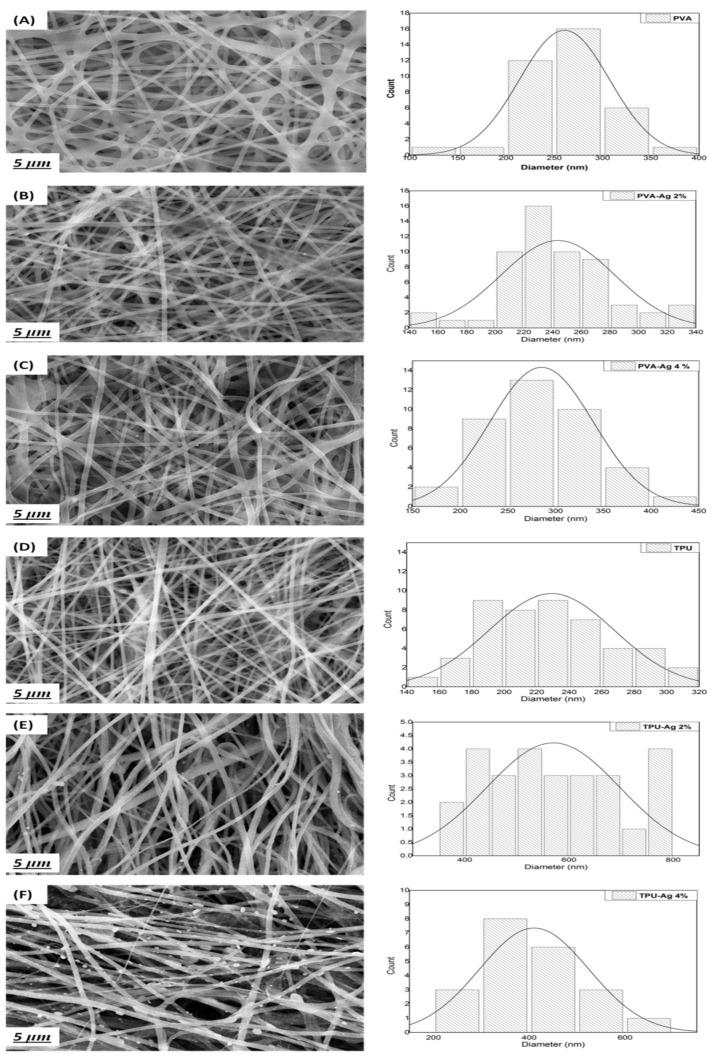
SEM images and fiber diameter distribution of PVA control (**A**), PVA-Ag 2% (**B**), PVA-Ag 4% (**C**), TPU control (**D**), TPU-Ag 2% (**E**), and TPU-Ag 4% (**F**).

Both PVA-Ag and TPU-Ag hybrid nanofibers morphology were examined by a field emission scanning electron microscope (FESEM). From the respective micrographic images, as shown in Figure 1, quantitative analysis of Fiber Diameter Distribution was obtained. Average fiber diameter of nanofibers for all samples was calculated through Image J as represented in Table 1. The images reveal the impact of Ag addition on the fiber formation and fiber morphology. As shown, homogenous distribution and beads-free fibers were obtained for all samples as a result of optimized spinning conditions and silver blending. The Ag impeded samples showed slightly larger fiber diameter due to the incorporation of Ag nanoparticles into the fiber structure.

### 3.2. Physicochemical Characterization of Nanofibers

#### 3.2.1. Fourier Transform Infrared Spectroscopy (FT-IR) Characterization

**Figure 2:**
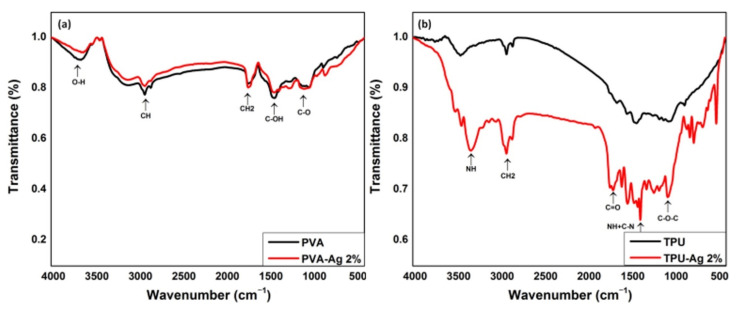
FT-IR of (**a**) PVA, PVA-Ag 2% and (**b**) TPU, TPU-Ag 2%, nanofiber.

The infrared spectra for the region 700–4000 cm^−1^ of prepared nanofibers of PVA and TPU with and without silver are shown in Figure 2. Peaks at 715, 871, 1091, 1431, 1670, 2923, and 3655 cm^−1^, as shown in Figure 2a, are characteristic peaks of PVA. Out of plane vibrations of both O–H and C–H bonds are responsible for the IR peaks at 715 and 871 cm^−1^, respectively. C–O stretching and bending vibrations of the CH_2_ group are responsible for the IR peaks at 1091 and 1431 cm^−1^; respectively. The C=O stretching vibrations of the remaining non-hydrolyzed vinyl acetate group of the PVA are responsible for an IR peak at 1737 cm^−1^. Stretching vibration of the CH_2_ group is responsible for an IR peak at 2923 cm^−1^. The OH stretching vibrations of strong hydrogen bonds from intra- and intermolecular form lead to the strong large peak at 3655 cm^−1^. The increase in transmittance of the silver-embedded PVA fibers was accompanied by a significant increase in the vibrational frequency dedicated to in-plane O–H vibrations with C–H vibrations at 3630 and 2922 cm^−1^. This shows how embedded silver interacts with the O–H group of PVA in the PVA fiber matrix. Out-of-plane O–H vibration is represented by the wide peak spanning at a lower wave number at 852 cm^−1^ [36].

On the other hand, as shown in Figure 2b, the pure TPU nanofiber N–H stretching band is observed at 3440 cm^−1^. The bands of the aliphatic CH stretching frequencies can also be seen at 2924 and 2858 cm^−1^. The N-H and C-H bond stretching frequencies were sharpened and moved to 3442 and 2856 cm^−1^, respectively [37]. The weakening of these bonds is suggested by the decrease in wavenumber of the N–H and C–H stretching frequencies, which may be due to an intermolecular interaction of the N–H with Ag. The following are the characteristic peaks of neat TPU nanofibers: 2950 cm^−1^ (CH_2_ asymmetric vibration); 1733 cm^−1^ (free C=O); 1689 cm^−1^ (C=O bond); 1599 cm^−1^ (urethane amide II); 1071 cm^−1^ O–C stretching of the hard segment; and 816 cm^−1^ C–O stretching of the soft segment (bending vibration in benzene ring). A wide transmittance band based at 3511 cm^−1^ was observed, which corresponded to –OH stretching vibration. The change in the fingerprint 400–1600 of these FTIR spectra could prove the Ag-incorporated TPU hybrid nanofibers and successful manufacturing.

#### 3.2.2. X-ray Diffraction of PVA, TPU, and Their Ag-Hybrid Nanofibers

**Figure 3:**
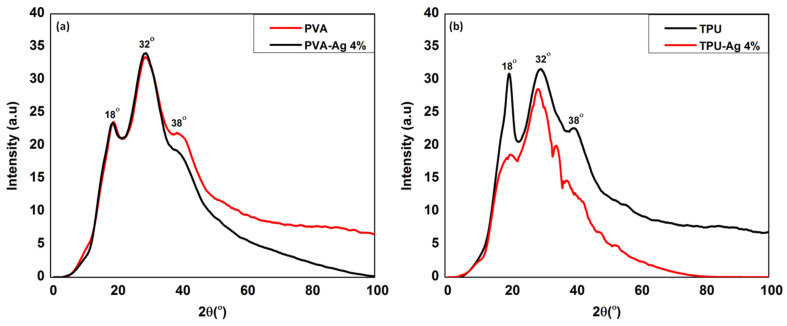
XRD of (**a**) PVA, PVA-Ag 4% and (**b**) TPU, TPU-Ag 4% nanofibers.

The XRD pattern for the synthesized fibers of PVA and TPU with and without silver nanoparticles is shown in Figure 3. The XRD pattern showed strong peaks from Braggs reflection at 2θ = 18°, 32°, and 38°, Figure 3a. The crystalline nature of the PVA polymer molecule is well understood to trigger the peaks at 2θ less than 20°. Strong intermolecular and intramolecular hydrogen bonding between the PVA chains can form the lattice planes corresponding to 2θ = 18°. The silver nanoparticles corresponding to the lattice plane are responsible for the X-ray diffraction peak at 38°. Spectroscopic characteristics of the silver nanoparticles is introduced in the file of supplementary data, Figures S1–S5, with particle size of Ag NPs to be 17–51 nm. The presence of intermolecular bonding between the PVA and the silver nanoparticles could explain the peaks at 32° [38].

The phase structures of the TPU-Ag hybrid nanofibers were also investigated using XRD analysis. Figure 3b shows the XRD diffractograms of the hybrid nanofibers. The XRD patterns exhibited that the TPU films containing Ag NPs were partly crystalline in nature, while the pristine TPU nanofibers were amorphous. As a result, during the electrospinning process, these Ag NPs were easily incorporated into the TPU nanofiber. XRD revealed diffraction peaks corresponding to the (111) and (200) planes of Ag in the TPU-Ag-in hybrid nanofibers, confirming the existence of Ag NPs in the TPU-Ag of nanofibers hybrid [39].

### 3.3. Antiviral Activity

Elechiguerra et al., [40] were the first to characterize metal nanoparticles antiviral activity, discovering that Ag NPs interact with HIV-1 in a size-dependent manner. They looked into the possibility that nanoparticle physicochemical properties are influenced by nanoparticle interactions with a capping agent molecule. Furthermore, Ag NPs inhibit the HIV-1 life cycle at the post-entry stage; silver ions may form complexes with electron donor groups containing sulfur, oxygen, or nitrogen that are normally present as thiols or phosphates on amino acids and nucleic acids, they may be able to block HIV-1 proteins. Viral infectivity assays showed that silver nanoparticles were veridical to HIV-1 [41]. Sun et al., [42] also investigated the viral inhibition of using Ag NPs. The interaction of Ag NPs with respiratory syncytial virus (RSV) virion particles was studied using TEM, and the results were intriguing. The Ag NPs appeared to interact with RSV, and were able to bind to the viral surface in a normal spatial arrangement.

Zhang and et al., [34] reported that 2–4% of Ag NPs concentration (*w*/*w*) showed a good antibacterial activity for both Gram-positive; *Staphylococcus aureus* (*S. aureus*) and Gram-negative *Escherichia coli* (*E. coli*) microorganisms on PVA nanofibers; moreover, Ag/PVA nanofibers are also found to contribute significantly to SERS with high sensitivity to 4-mercaptophenol (4-MPh) molecules.

**Figure 4:**
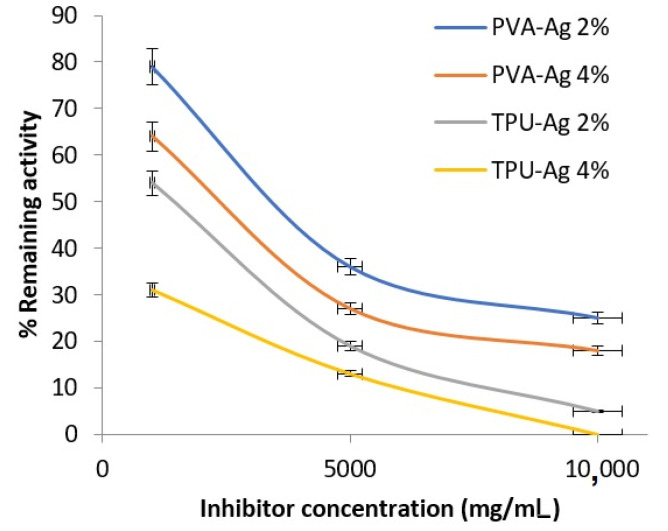
Percentage of remaining activity of HIV-1 Protease in presence of the tested nanofibers.

In our study, the antiviral activity of PVA-Ag (PVA, PVA-Ag 2% and PVA-Ag 4%) and TPU-Ag (TPU, TPU-Ag 2% and TPU-Ag 4%) was evaluated against the most predominant RNA viruses. Data in Figure 4 revealed that, the percentage of the relative activity is inversely proportion with log inhibitor concentration which means that by increasing the Ag concentration, the HIV-1 protease activity was decreased. It was found that TPU-Ag was more effective than PVA-Ag in both tested Ag concentrations. The interaction between HIV particles and Ag NPs is apparently due to the Ag NPs that are able to bind to the virus. The sulfur-bearing residues of the glycoprotein knobs were discovered to be the most likely sites for interaction. Due to their essentially free surface area, the Ag NPs released from the polymer matrix to demonstrate a high inhibitory effect. Authors could attribute the higher pronounced inhibitory effect of TPU rather than that of PVA of silver nanohybrids to the particle distribution of the Ag nanoparticles inside the caves of the polymeric matrix of the TPU in a way that may permit a kind of interaction of Ag NPs with S-protein of the virus and consequently it causes more deactivation than that of the PVA. Moreover, solubility of the PVA could decrease such interaction with respect to the insoluble TPU fibers.

**Figure 5:**
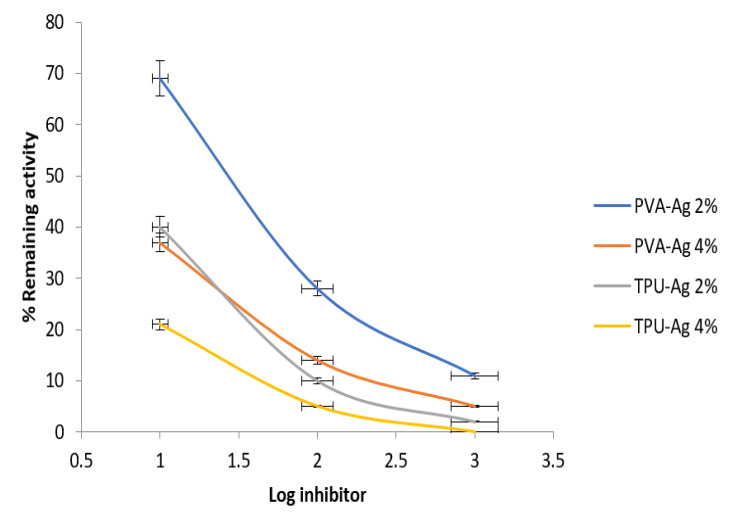
Percentage of remaining activity of blocking viral fusion of SARS-COV-2.

On the other hand, in order to evaluate the antiCOVID-19 effect of manufactured PVA-Ag and TPU-Ag, a SARS-CoV-2 Inhibitor Screening Kit was used. Data in Figure 5 confirmed the effectiveness of the proposed nanofibers in deactivation of the spike protein of SARS-Cov-2. It was revealed that Ag concentration is the most effective factor in the antiviral activity. TPU was found to be more effective than PVA in Ag loading and the corresponding activity.

It was recently reported [43] that nanoparticles or polymers had little or no antiviral activity on their own, but that a hybrid of both might have a lower effective viral titer. This may be due to the hybrid substance property of being a combination of modified materials rather than a hybrid of two materials in their original state. Antiviral activity was attributed to the presence of ions produced on the surface of nanoparticles. The presence of reactive oxygen species in Ag NPs may produce ions that disrupt the lipid bilayer membrane’s structural integrity or the virion particles surface antigens. The virus particles’ ability to interact with the cell surface receptor required for attachment and subsequent entry into the host cell should be harmed as a result of this interference [44].

### 3.4. Antibacterial Activity

**Figure 6:**
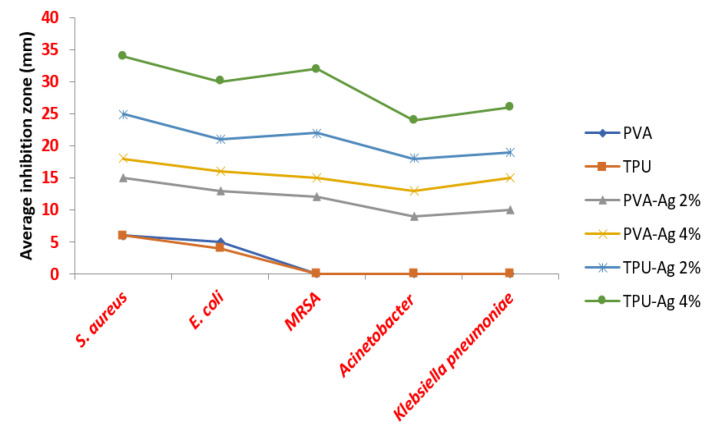
Relationship between average inhibition zone and Ag NPs loading concentration on PVA and TPU nanofibers.

Silver nanoparticles have a wide range of applications, including wound dressings, medical device coatings, and Ag NP-impregnated textile fabrics [45]. The advantage of impregnation with Ag NPs is that silver ions are released continuously, increasing antimicrobial effectiveness. In our study, in order to investigate the antibacterial activity of PVA-Ag and TPU-Ag, different bacterial strains were explored by measuring the inhibition halo around the tested nanofiber. The nanofibers loaded with silver showed higher antibacterial effect against Gram positive bacteria. The antibacterial effect is attributed to the small size of the hybrid fibers, which allows them to be absorbed by hydrogen bonding force and static electricity on the bacterial cell walls, resulting in inhibitory effect with blocking of the bacterial growth, eventually leading to their death, Figure 6 and Table 2 [24].

**Figure 7:**
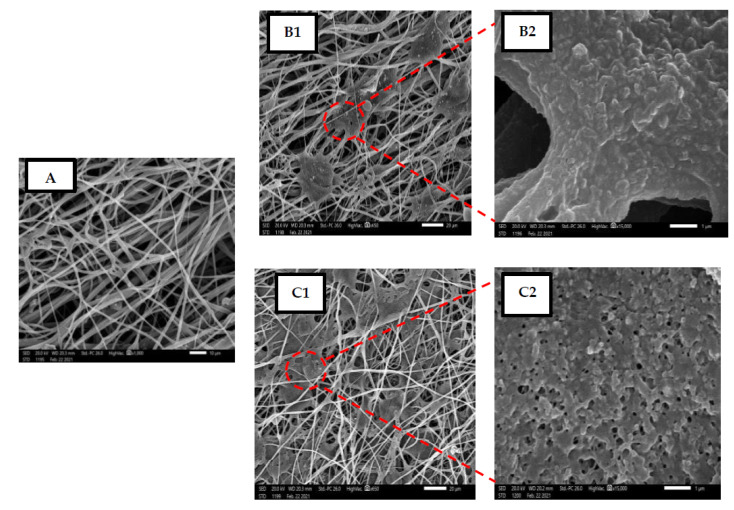
SEM image of the bacterial cell death in matrix of manufactured nanofibers where (**A**): Control untreated fibers, (**B1**): PVA-Ag 4%, (**B2**): Zoom of (**B1**,**C1**): TPU-Ag 4%, (**C2**): Zoom of (**C1**).

TPU-Ag was found to have a superior activity than PVA-Ag and by increasing Ag concentration from 2% to 4%, the activity increased. Hence, TPU-Ag nanofibers were subjected to further evaluation using SEM study to indicate their bactericidal mechanism. It was found that the tested bacteria were adsorbed on the TPU-Ag nanofibers surface which led to lysis of the bacterial cells and releasing of cell contents. Both images and observed activities of Ag may elucidate a mechanism of Ag entry into bacterial cells via diffusion and osmosis, destroying cell enzymes and proteases, followed by bacterial cell destruction. The smaller the size of Ag NPs, the easier for them to get inside the bacterial cell and, consequently, destroy them (see Figure 7) [25].

According to Abdelrahman et al. [46], Ag NPs could be gradually released from the polymer matrix of PVA due to its swelling property, and demonstrated superior antibacterial activities. Although the exact antibiosis theory of Ag nanomaterials is still debated in the field of bacterial toxicology, Ag NPs are adsorbed on the cell membrane’s surface and can disrupt normal cell functions such as respiration and permeability. Secondly, Ag NPs can enter bacteria and continue to kill them by interacting with phosphorus and sulfur-containing compounds like DNA and active enzyme. Finally, Ag NPs release Ag^+^ ions, which contribute significantly to the antibacterial properties [47]. In terms of the differences in bactericidal activities caused by different shapes of Ag NPs, it has been proven that silver’s reactivity is due to high atom density facets [48]. Moreover, the silver mechanism of action is thought to be based on Ag ions, which suppress bacterial growth by inhibiting respiratory enzymes and electron transport components, as well as interfering with DNA functions [49].

### 3.5. Safety of the Proposed PPE

Based on detected antiviral and antibacterial efficacy of the manufactured membranes, authors could recommend using them as effective PPEs; surgical gowns, gloves, overshoes, haircaps, etc.; for a single-use to prevent spreading of viruses and bacteria among healthcare workers taking into consideration the guidelines of the World Health Organization. Through the proposed methodology, embedding of AgNPs in the polymer matrices (PVA or TPU) could help in tuning of metal release properties, and minimizing the risk of nanoparticle release or leakage to user’s skin [50].

Comparative studies demonstrated that inclusion of AgNPs in skin topical antibacterial agents is safe, has negligible effects on human health if used in reasonable quantities and is considered an excellent wound dressing as AgNPs-loaded hydrogel [25]. Cytotoxicity and genotoxicity of AgNPs were observed at concentrations much higher than MIC of AgNPs [51]. Furthermore, transcutaneous passage of AgNPs through human skin, did not penetrate healthy intact human skin, did not easily penetrate the skin barrier, and had no detrimental effects on keratinocytes and dermal fibroblasts [52]. Thus, AgNPs possess the outstanding potential for use in wound dressing applications. A controlled, cross over time exposure (3, 7, and 14 days) study of orally dosed (10 ppm) commercial AgNPs (5–10 nm) demonstrated the absence of any changes in human metabolic, hematologic, urine, and physical findings or imaging morphology [53].

On contrary, there is considerable evidence for the significant transdermal penetration of AgNPs into capillaries during the use of surgical dressings, textiles, and cosmetics [54,55]. A correlation between dermal exposure, tissue accumulation of AgNPs (100 nm), and dose-dependent histopathological abnormalities in the skin, was suggested and all of which was exhibited by a reduced thickness of the papillary layer and the epidermis [56]. Also, AgNPs applied as nanocrystalline silver dressing for four-to-six days to human skin, can penetrate beyond the stratum corneum and reach as deep as the reticular dermis [24].

## 4. Conclusions

Nanofibers of PVA and TPU with Ag NPs concentrations from 2% to 4% blends were manufactured properly by electrospinning. The optimized spinning conditions and silver blending were implemented and homogenous distribution and beads-free nanofibers in all samples were achieved. Screening of both antiviral and antibacterial activities of PVA-Ag and TPU-Ag against different viruses including COVID 19 and some bacterial strains revealed that, TPU is more active than PVA in all Ag loadings as SARS-Cov-2 antiviral. TPU-Ag is more effective than PVA-Ag in all Ag concentration as HIV-1 antiviral hybrid as well as antibacterial potential activity. Antibacterial mechanism of TPU-Ag nanofibers shown by SEM disclosed that the tested bacteria adsorbed on TPU-Ag nanofibers’ surface and subsequent lysis effect. Such recorded activities are directly proportional to the Ag concentration. As the current study introduces manufactured NFs having good physical and mechanical properties with both antiviral and antibacterial potentials against SARS-Cov-2, it can be recommended that using such nanofibers as potential PPE could help decrease the spread of coronavirus and bacterial contamination particularly for healthcare professionals in their workplace settings.

## Figures and Tables

**Table 1:** Average fiber diameter distribution of PVA and TPU nanofibers.

Sample	PVA	PVA-Ag 2%	PVA-Ag 4%	TPU	TPU-Ag 2%	TPU/Ag 4%
Average Fiber Diameter (nm)	250 ± 10	240 ± 7	230 ± 12	280 ± 10	550 ± 30	400 ± 17

**Table 2:** The average inhibition zone with the Ag loading concentration on PVA and TPU nanofibers.

	Inhibition Zone Diameter (mm ± SDV *)									
Nanofiber	*S. aureus*		*E. coli*		*MRSA*		*Acinetobacter*		*Klebsiella-Pneumoniae*	
PVA	6	±0.58	5	±0.58	0	±0.00	0	±0.00	0	±0.00
TPU	6	±0.58	4	±0.00	0	±0.00	0	±0.00	0	±0.00
PVA-Ag 2%	15	±1.53	13	±1.53	12	±1.00	9	±0.58	10	±0.58
PVA-Ag 4%	18	±1.00	16	±1.53	15	±0.58	13	±0.58	15	±0.58
TPU-Ag 2%	25	±2.52	21	±0.58	22	±0.58	18	±0.58	19	±0.00
TPU-Ag 4%	34	±2.08	30	±1.15	32	±1.15	24	±1.15	26	±0.58

* The values are expressed as mean ± SDV.

## Data Availability

The data presented in this study are available on request from the corresponding author.

## Supplementary Materials

The following are available online at https://www.mdpi.com/article/10.3390/polym13111776/s1. Figure S1: UV-Vis absorption spectrum of silver Nanoparticles. Figure S2: Transmission Electron Microscope (TEM) image of AgNPs. Figure S3: SEM of Ag NPs. Figure S4: XRD pattern of Ag NPs. Figure S5: FTIR spectra of the Ag NPs. Figure S6: SEM images and Segmented images of PVA control (A), PVA-Ag 2% (B), PVA-Ag 4% (C), TPU control (D), TPU-Ag 2% (E), and TPU-Ag 4% (F).
