# Supplementary material for: Hybrid Nanofibrous Membranes as a Promising Functional Layer for Personal Protection Equipment: Manufacturing and Antiviral/Antibacterial Assessments
Source: Polymers (Basel). 2021 May 28;13(11):1776. doi: 10.3390/polym13111776 (PMC8198978; doi:10.3390/polym13111776)
Supplement: Supplementary file 1 [file polymers-13-01776-s001.zip › polymers-1215287-SI.pdf]

Supplementary Data

Hybrid Nanofibrous Membranes as a Promising Functional Layer for Personal Protection Equipment: Manufacturing and Antiviral /Antibacterial Assessments

Latifah Abdullah Alshabanah [1], Mohamed Hagar [2,3*], Laila A. Al-Mutabagani [1], Ghada M. Abozaid [4], Salwa M. AbdAllah [5], Nader Shehata [6, 7, 8, 9], Hoda Ahmed [2,10], Ahmed H. Hassanin [6, 11, 12]

1. Chemistry Department, College of Science, Princess Nourah bint Abdulrahman University, Riyadh 11671, Saudi Arabia; laalsabanah@pnu.edu.sa (L.A.A.), laalmutbagani@pnu.edu.sa (L.A.A.-M.),
2. Chemistry Department, College of Sciences, Yanbu, Taibah University, Yanbu 30799, Saudi Arabia
3. Chemistry Department, Faculty of Science, Alexandria University, Alexandria 21321, Egypt
4. Pharmaceutical practice department, college of pharmacy, Princess Nourah bint Abdulrahman University, Riyadh 11671, Saudi Arabia, Gaabozeed@pnu.edu.sa (G. M).
5. Mammalian and Aquatic Toxicology Department, Central Agricultural Pesticides Lab (CAPL), Agricultural research Center (ARC), Giza, Egypt.
6. Center of Smart Materials Nanotechnology and Photonics (CSMNP), SmartCI Research Center, Alexandria University, Alexandria 21544, Egypt
7. Department of Engineering Mathematics and Physics, Faculty of Engineering, Alexandria University, Alexandria 21544, Egypt
8. USTAR Bio innovations Center, Faculty of Science, Utah State University, Logan, Utah 84341, United States
9. Kuwait College of Science and Technology (KCST), Doha District 13133, Kuwait
10. Department of Chemistry, Faculty of Science, Cairo University, Cairo 12613, Egypt.
11. Materials Science & Engineering Department, School of Innovative Design Engineering, Egypt-Japan University of Science and Technology (E-JUST), New Borg El-Arab City, Alexandria 21934, Egypt
12. Department of Textile Engineering, Faculty of Engineering, Alexandria University, Alexandria 21544, Egypt

*Correspondence: Mohamed Hagar; mhagar@taibahu.edu.sa

Structural Analysis of AgNPs using XRD

UV-Vis Spectrophotometer Analysis

UV-Vis spectroscopy is a method that used to examine the production of the nanoparticles based on their optical properties (Kumar et al., 2013). Absorption spectrum of silver nanoparticles shows strong absorption at 425 nm (Figure1S).

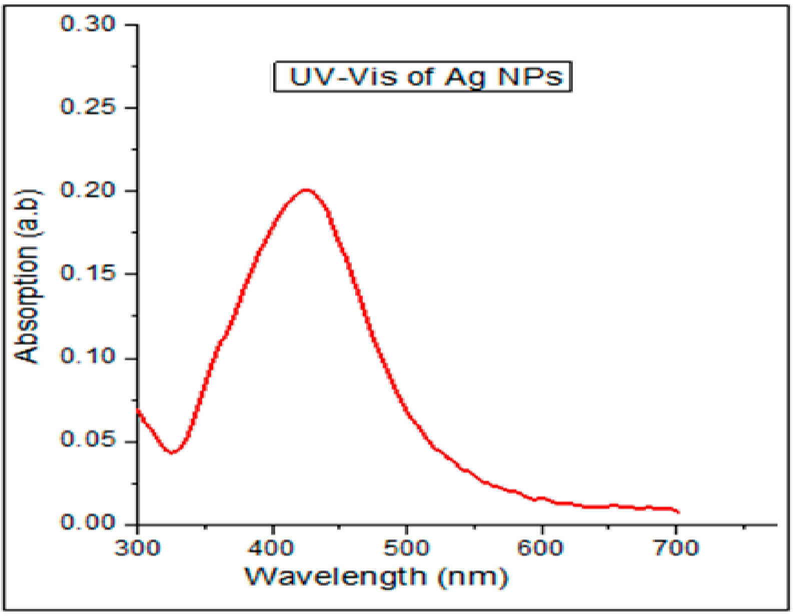

Figure 1S: UV-Vis absorption spectrum of silver Nanoparticles.

Transmission Electron Microscope Images (TME)

The typical TEM micrographs of the synthesized Ag nanoparticles are presented in Figure (2S). It is observed that most of the Ag nanoparticles were spherical in shape and homogeneously distributed, Figure (2S).

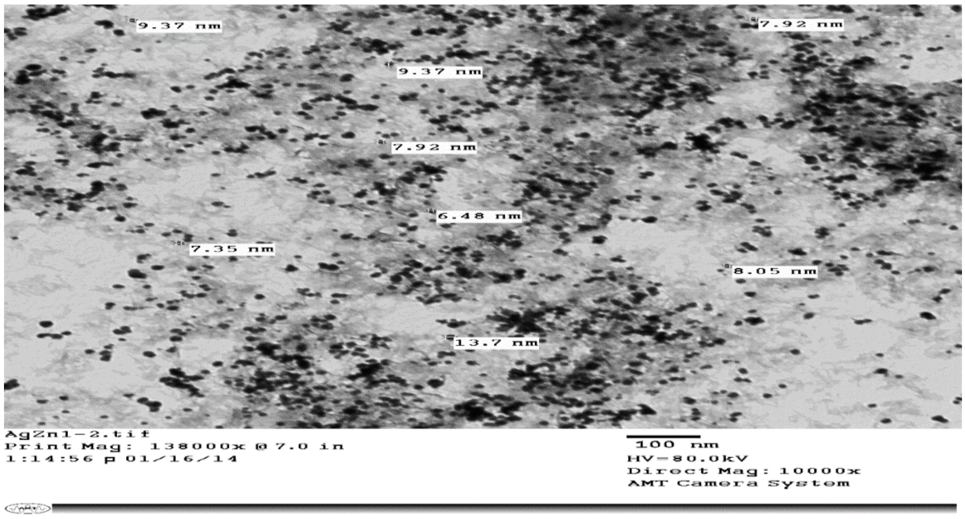

Figure (2S): Transmission Electron Microscope (TEM) image of AgNPs.

Scanning Electron Microscopy (SEM):

The SEM images of Ag nanoparticles are shown in Figure (3S). The morphologies of Ag NPs show that the particles ate spherical in shape in the range of 17- 51 nm .

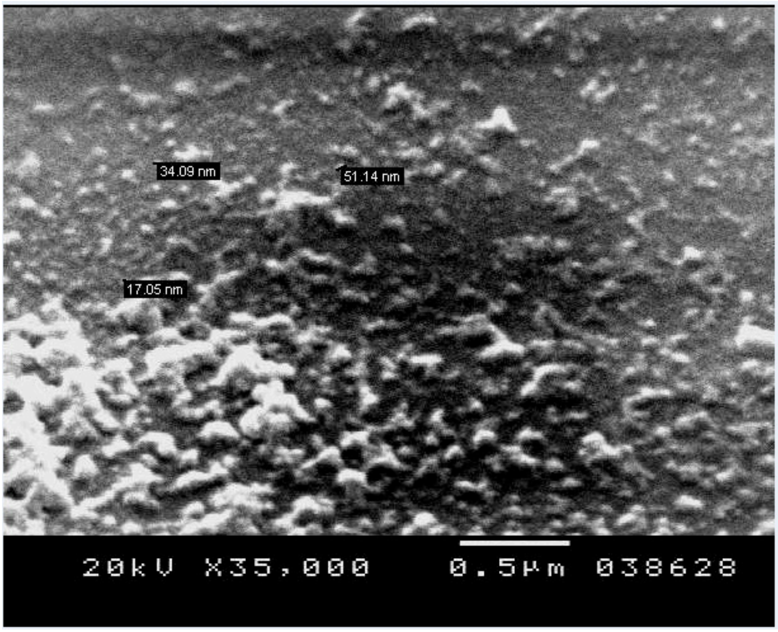

Figure (3S): SEM of Ag NPs.

X-ray Diffractometer Analysis:

Figure 4S shows XRD data of the Ag nanoparticles. The peaks at 2θ = 38.07°,44.18°, 64.37° and 77.29° can be assigned to reflections from the (1 1 1), (2 0 0), (2 2 0) and (3 1 1) planes respectively, of metallic silver in FCC phase.

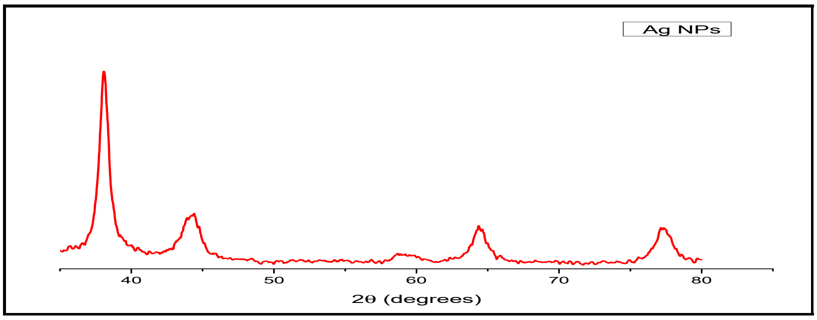

Figure (4S) XRD pattern of Ag NPs

FT-IR spectroscopy Analysis:

Figure (5S) shows the FTIR spectrum recorded for silver nanoparticles. The spectrum for the silver nanoparticles shows various peaks. The peaks at 3419 and 1629 cm-1 are very broad and strong, and can be assigned to the hydroxyl groups. The band at 1648.7 indicates the formation of metal carbonyl groups. It is due to the stabilization of Ag nanoparticles by the –coo- group of trisodium citrate. Another peak is observed at 1384 cm-1 which was concluded to be due to the nitrate ions.

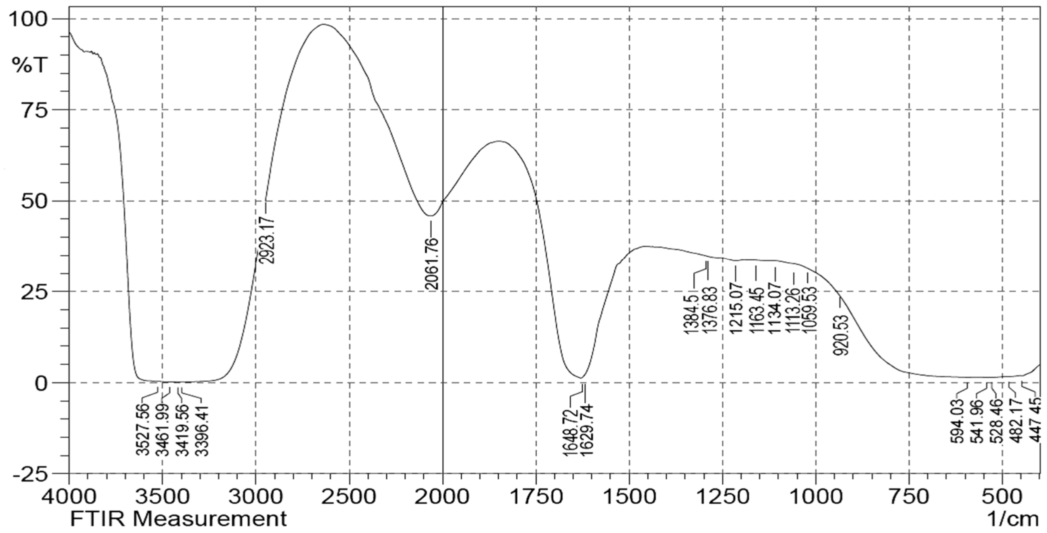

Figure (5S): FTIR spectra of the Ag NPs

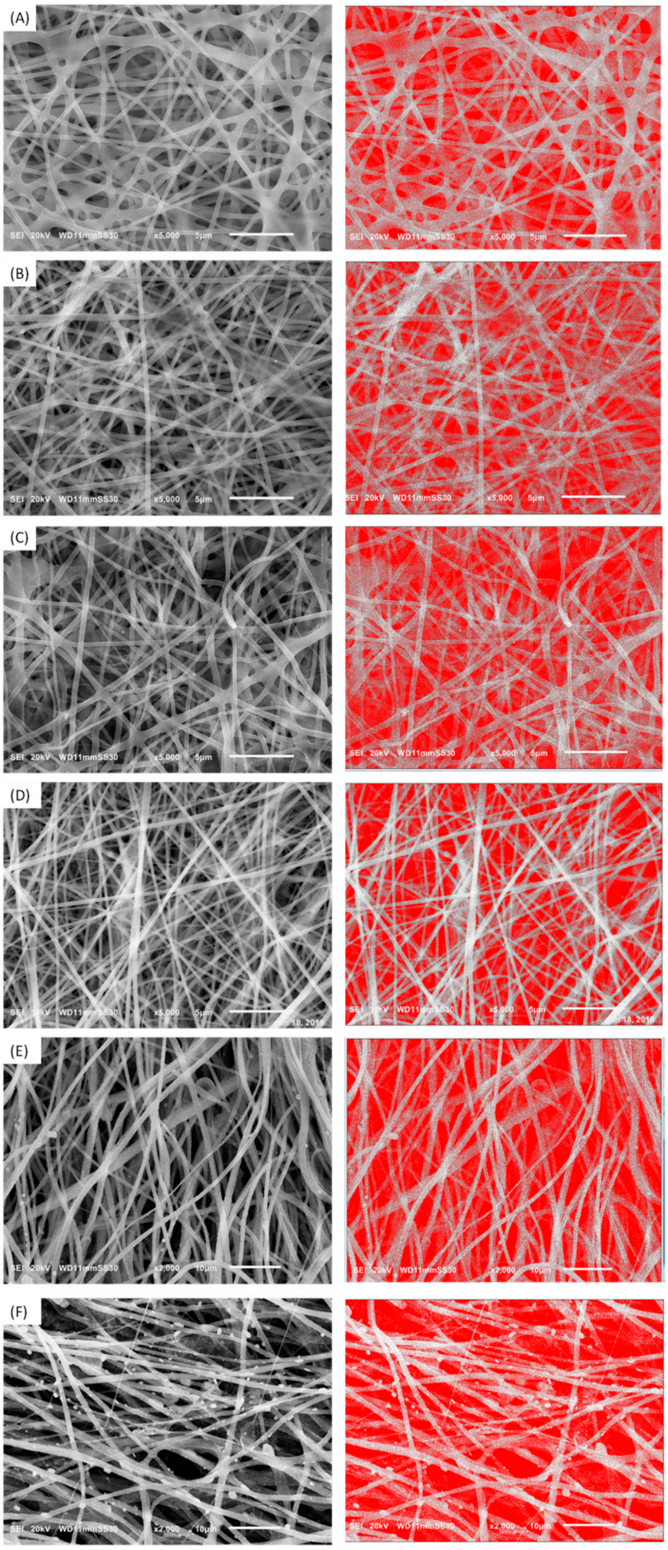

Figure S6: SEM images and Segmented images of PVA control (A), PVA-Ag 2% (B), PVA-Ag 4% (C), TPU control (D), TPU-Ag 2 % (E), and TPU-Ag 4% (F)

References

Kumar, Surabhi Siva, Putcha Venkateswarlu, Vanka Ranga Rao, and Gollapalli Nagewsara Rao. 2013. "Synthesis, Characterization and Optical Properties of Zinc Oxide Nanoparticles." *International Nano Letters* 3 (**1**): 30-34.
